# Unraveling the Conversion Evolution on Solid‐State Na–SeS_2_ Battery via In Situ TEM

**DOI:** 10.1002/advs.202200744

**Published:** 2022-03-23

**Authors:** Ziqi Zhang, Zaifa Wang, Long Zhang, Di Liu, Chuang Yu, Xinlin Yan, Jia Xie, Jianyu Huang

**Affiliations:** ^1^ Clean Nano Energy Center State Key Laboratory of Metastable Materials Science and Technology Yanshan University Qinhuangdao Hebei 066004 China; ^2^ State Key Laboratory of Advanced Electromagnetic Engineering and Technology School of Electrical and Electronic Engineering Huazhong University of Science and Technology Wuhan 430074 P. R. China; ^3^ Institute of Solid State Physics Vienna University of Technology Vienna 1040 Austria

**Keywords:** composite cathode, in situ TEM, Na–S battery, Se doping, solid‐state batteries

## Abstract

All‐solid‐state (ASS) Na–S batteries are promising for a large‐scale energy‐storage system owing to numerous merits. However, the high conversion reaction barrier impedes their practical application. In this work, the basic mechanism on how Se catalyzes the conversion reaction in the Na–S batteries is unraveled. The sodiation/desodiation of Na–SeS_2_ nanobatteries are systematically evaluated via in situ transmission electron microscopy (in situ TEM) with a microheating device. The real‐time analyses reveal an amorphous Na–Se*_x_* S*_y_* intermediate phase appears during the direct conversion from SeS_2_ to Na_2_S, and a reverse reaction succeeds at 100 °C with a prior formation of Se. The absence of polysulfides and a much lower desodiation temperature in contrast to Na–S nanobatteries demonstrate that the Se incorporation significantly lowers the conversion reaction barrier. According to these findings, the ASS SeS_2_ batteries using a Na_3_SbS_4_ solid electrolyte (SE) are assembled using various SE:C ratios in the composite cathodes to investigate the effect of the ion and electron transport on the electrochemical properties, including the effective transport properties, MacMullin number, and the tortuosity factor. The obtained results in turn confirm the findings from the in situ TEM. These findings are applicable to optimize other S‐based active materials and improve their utilization.

## Graphical abstract

Se incorporation lowers the energy barrier in a Na–S battery and thereby catalyzes the reversible conversion reaction. The real‐time transmission electron microscopy discovers an amorphous Na–Se_*x*_S_*y*_ intermediate, promoting the transformation from SeS_2_ to Na_2_S without the formation of polysulfides. The reverse desodiation accomplishes at a low temperature of 100 °C.

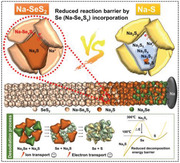

## 1 Introduction

The full utilization of intermittent renewable energies requires large‐scale energy storage systems with high safety and reliability.^[^ ^1^ ^]^ Na–S batteries with merits of abundant raw materials and high energy densities are very promising for such application.^[^ ^2^ ^]^ Typically, Na–S batteries work at room temperature with a higher specific capacity and lower energy consumption compared to their high temperature counterparts are highly anticipated.^[^ ^3^ ^]^ Nevertheless, similar to the Li–S batteries, the Na–S batteries also suffer the polysulfide shuttle effect, which causes the bottleneck issue regarding the irreversible capacity loss and rapid capacity degradation during electrochemical cycling. One of the methods to eliminate the shuttle effect is to use ASS Na–S batteries with a SE as the ion conductor.^[^ ^4^ ^]^

Another drawback of the Na–S batteries is the low electronic and ionic conductivities within the elemental S active material, especially in ASS batteries using SE with solid–solid point contacts. The intrinsic modification and the optimization of cathode components are required for properly utilizing S. Accordingly, high conductivity elements such as Fe, Ti, Se, Te are usually introduced for S to improve the electrochemical activity.^[^ ^5^ ^]^ Among them, Se is particularly attractive, not only because Se and S are in the same group in the periodic table, but also because Se doping effectively accelerates the reaction dynamics of S, and alleviates the formation of polysulfides, as is extensively studied in the liquid‐electrolyte‐based batteries.^[^ ^6^ ^]^ For various Se–S ratios,^[^ ^7^ ^]^ SeS_2_ gains a high ionic conductivity and decent electronic conductivity, thereby an optimal composition for ASS Li–S batteries with good rate performance. In addition, SeS_2_ has a high theoretical capacity of 1342 mAh g^−1^.^[^ ^8^ ^]^

However, the fundamental science for the role of Se in Na–S system is still unclear so far. One of the reasons lies in the limitation of the characterization method due to the moisture sensitive nature of Na‐ and S‐based products. In view of this point, real‐time characterizations such as, in situ Raman, X‐ray diffraction (XRD), transmission electron microscopy (TEM) etc., filled with inert protective atmosphere are particularly suitable for evaluating ASS Na–S batteries to provide valuable and reliable information.^[^ ^9^ ^]^ In situ TEM equipped with charging/discharging device can not only monitor the real‐time phase transition during the electrochemical processes, but also analyze the reaction kinetics by calculating the migration rate of the reaction front (RF).^[^ ^10^ ^]^ Generally, various carbon materials such as spherical mesoporous carbon, grapheme, and carbon nanotube were used as S/Se carrier for carrying out the electrochemical processes.^[^ ^11^ ^]^ The in situ TEM studies on the charging/discharging process of the ASS Na–S nanobatteries reveal multistep sodiation transformations from S to Na_2_S via polysulfides (Na_2_S_5_, Na_2_S_4_, Na_2_S_2_), and the Na_2_S sodiation product cannot be reversibly oxidized until ≈300 °C.^[^ ^12^ ^]^ The discovery of the intermediate phases and the evaluation of the RF migration are important to understand the redox reaction kinetics during the electrochemical processes.

The component optimization in the cathode is significant for realizing high capacity and good rate performance for ASS batteries. The quantification of the effective charge transport and the tortuosity factor have been used to assess the ion/electron transport bottleneck in an electrode for the batteries using liquid organic electrolyte, and thereby optimizing the component design of the electrode.^[^ ^13^ ^]^ Such an optimization is more important in ASS batteries due to the heterogeneous three‐phase disperse system in the composite cathode, especially for S‐based active materials with low electron and ion conductivities that need carbon and SE conducting additives to provide sufficient electron/ion transport.^[^ ^14^ ^]^ In addition to a proper compositional proportion, the corresponding quantitative evaluation of ion/electron transport in the composite cathode is worth to make clear, which is also a key factor to improve the battery properties.^[^ ^15^ ^]^

In this work, a study of in situ TEM implemented with nanobatteries gives the fundamental insight into understanding the structural and chemical evolutions on the ASS Na–SeS_2_ batteries and reveals the significant influence of Se incorporation in the S‐based active material. We use SeS_2_ rather than Se*_a_* S_1‐_ *_a_* to avoid elemental Se and S and other impurities. The amorphous Na–Se*_x_* S*_y_* intermediate phase generated during the conversion reaction is observed for the first time, and demonstrates a notable feature to lower the reaction energy barrier. Moreover, the comprehensive in situ TEM investigation figures out the dominant factor of the electrochemical conversion in the Na–SeS_2_ nanobatteries. The TEM findings guide the instruction toward the component design of the cathode. The ASS Na–SeS_2_ battery with an optimized composite cathode delivers a high reversible capacity and excellent rate capability. The corresponding evaluation of the reaction kinetics through the galvanostatic intermittent titration technique (GITT) and in situ electrochemical impedance spectra (EIS) in turn verify the TEM result, demonstrating a reasonable optimization strategy regarding Na–S batteries.

## 2 Results and Discussion

**Figure 1:**
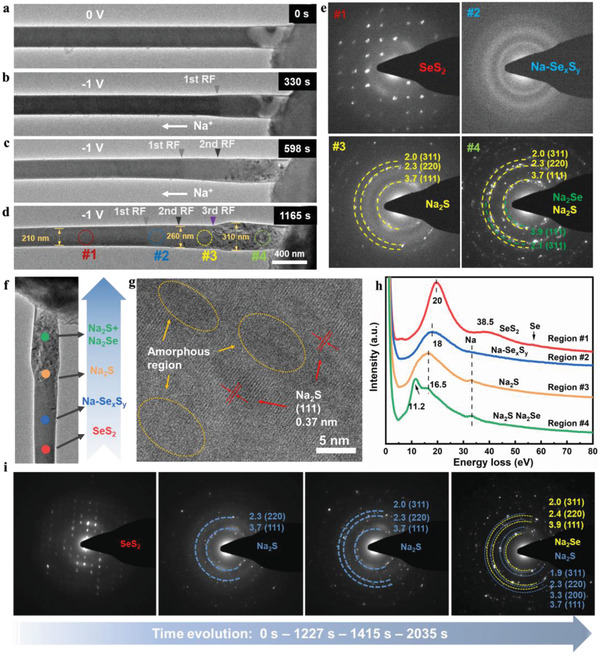
a–d) Time‐elapsed structural evolution of SeS_2_@CNT upon sodiation under a bias voltage of −1 V. e) EDPs at region #1‐4 labeled in d). f) Summary of the sodiation procedure of SeS_2_@CNT. g) HRTEM image at region #3. h) EELS profiles at region #1‐4. i) Operando EDP measurements for SeS_2_@CNT upon sodiation.

The nanobatteries for in situ TEM observations were constructed using the carbon‐nanotube‐encapsulated SeS_2_ (SeS_2_@CNT, for both single‐crystalline and amorphous SeS_2_) as cathode. **Figure** **1**a–d displays the time‐resolved structure evolutions of SeS_2_@CNT (single‐crystalline SeS_2_) during the sodiation process at room temperature. When SeS_2_@CNT was attached initially with Na, there is no reaction on the contact surface (Figure 1a). When a bias voltage of −1 V was applied for sodiation for 330 s (Figure 1b), the 1st RF is obviously observed, which was marked with a gray triangle. The reaction occurs perpendicular to the CNT, so the electron transfer takes place along both CNT and SeS_2_, but is blocked by the Na_2_O layer that serves as an ion‐conducting electrolyte. When Na^+^ continued to migrate for another 268 s (in total 598 s), the 2nd RF is observed (marked with a black triangle), indicating the formation of new phases, as shown in Figure 1c. At 1165 s, the 3rd RF (marked with a purple triangle, Figure 1d) starts to generate, and the diameter expansion of SeS_2_@CNT can be distinctly observed. The diameters of the three selected regions representing before, partial, and complete sodiation were measured, respectively, as 210, 260, and 310 nm, as shown in Figure 1d. The completion of sodiation is judged from two aspects. First, the diameter of the CNT no longer changes. Second, the products are determined to Na_2_S and Na_2_Se. The volume expansions of SeS_2_ are calculated to be 53% and 118%, which are smaller than the theoretical value of 170% due to the confinement of the CNT. The diameter variation and volume expansion of various SeS_2_ for the nanobatteries at different voltages are summarized in Table S1 (Supporting Information).

The selected area electron diffraction patterns (EDPs) were collected at four different regions from intact to fully reacted (sodiation), marked with red (#1), blue (#2), yellow (#3), and green (#4) circles, as illustrated in Figure 1e. In region #1, single‐crystalline SeS_2_ is identified from the EDP. Remarkably, in region #2, an amorphous instead of crystalline phase was probed, as indicated by the amorphous halos. This demonstrates that an amorphous Na–Se–S intermediate phase first forms during the sodiation, which is designated as Na–Se*_x_* S*_y_*, though its detailed composition is unknown yet. Region #3 coexists amorphous and crystalline phases. The diffraction rings are associated with (111), (220), and (311) of Na_2_S, indicating that the first formed crystalline product is Na_2_S. This is different from the previous result that Na_2_Se first generated in the Na–SeS_2_ batteries using liquid organic electrolyte.^[^ ^16^ ^]^ The EDP analysis at region #4 exhibits that (111) and (311) diffraction rings belong to Na_2_Se appear in addition to Na_2_S. Hence, the discharging of single‐crystalline SeS_2_ is supposed to follow the sequence of SeS_2_→Na–Se*_x_* S*_y_* →Na_2_S→Na_2_S/Na_2_Se (Figure 1f).

To ensure the reliability and generalizability of the TEM result, the reproducible experiment using amorphous replacing crystalline SeS_2_ as an active material was carried out in the same test conditions, as shown in Figure S1 (Supporting Information). The resulting products of an amorphous SeS_2_ during the sodiation process are the same as those of single‐crystalline SeS_2_, demonstrating a generalizability of the phase evolutions during the sodiation process in Na–SeS_2_ batteries, regardless of the crystallinity of SeS_2_. The high‐resolution transmission electron microscope (HRTEM) image on region #3 displays only the amorphous phase and crystalline Na_2_S, as shown in Figure 1g. The distance between two fringes (Figure S2, Supporting Information) is 0.37 nm, consistent with the (111) plane of Na_2_S. No sodium polysulfides is observed. The HRTEM result confirms that the crystalline Na_2_S was generated directly from the amorphous Na–Se*_x_* S*_y_* phase.

The electron energy loss spectroscopy (EELS) were conducted at these four regions to distinguish the element variation during the different sodiation stages, as shown in Figure 1h. The plasmon oscillation peaks of SeS_2_ at 20 and 38.5 eV are identified from the spectrum of region #1 (Figure 1d). By increasing Na proportion during sodiation, the main peak at 20 eV shifts to 18 eV in region #2 and then to 16.5 eV in region #3, confirming a continuous phase transition. The peaks at 11.2 and 16.5 eV in region #4 are ascribed to the final products of Na_2_S and Na_2_Se. The peak at 33.2 eV is attributed to Na.

To monitor the evolution process during sodiation at a fixed position, operando EDPs (Figure 1i) were performed to investigate the successive process without suspension, which can reflect the real‐time SeS_2_ evolution states. The single crystalline SeS_2_ evolved to amorphous Na–Se*_x_* S*_y_* at the beginning of sodiation. Then the amorphous phase directly generated the crystalline Na_2_S. The sodiation eventually forms the crystalline Na_2_Se in addition to Na_2_S. The operando observations are in accordance with the results from the in situ measurements on the four selected regions shown in Figure 1d. The repeatable results are captured from another set of operando EDPs, as displayed in Figure S3 (Supporting Information). In brief, only one intermediate transition exists in Na–SeS_2_ nanobattery, i.e., SeS_2_→Na–Se*_x_* S*_y_* →Na_2_S and finally Na_2_S/Na_2_Se. This is significantly different from the sodiation process in Na–S nanobatteries, which exists step‐by‐step transitions with the intermediate phases including Na_2_S_5_, Na_2_S_4_, and Na_2_S_2_.^[^ ^12^ ^]^ The different scenarios indicate a lower sodiation‐reaction‐energy barrier for SeS_2_, which can be attributed to the formation of Na–Se*_x_* S*_y_* by Se incorporation.

**Figure 2:**
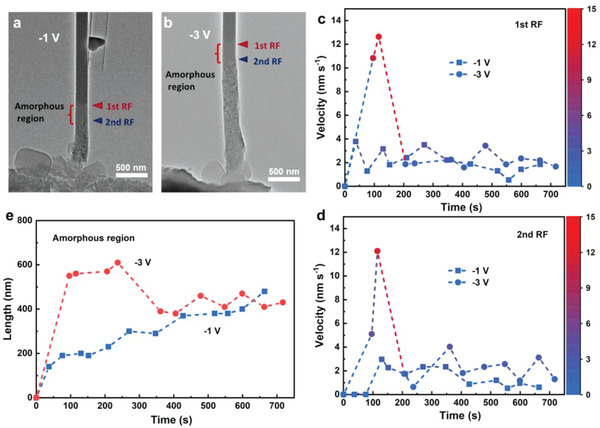
a,b) TEM images illustrating the 1st and 2nd reaction fronts and the corresponding amorphous regions for the nanobatteries by applying −1 and −3 V bias voltages. c,d) Migration velocity of the 1st c) and 2nd d) RFs as a function of reaction time at −1 and −3 V. e) Length of the amorphous region.

To evaluate the different influences of the ion and electron transport on the redox reaction of the Na–SeS_2_ nanobattery, the reaction kinetics were investigated systematically with the in situ TEM technique for the sodiation and desodiation processes. First, the sodiation was performed at different bias voltages of −1 and −3 V, respectively, as shown in **Figure** **2**a,b. The length of the amorphous region (Na–Se*_x_* S*_y_*) is measured from the distance between the 1st and 2nd RF. The velocity of the RF migration can be used to evaluate the sodiation reaction kinetics. Figure 2c,d, respectively, shows the velocity of the 1st and 2nd RF as a function of reaction time. During the initial 200 s, the increased bias voltage at −3 V obviously improves the migration speed of the 1st and 2nd RF. Figure S4 (Supporting Information) displays the *I*–*V* curve applied on the Na–SeS2@CNT nanobatteries. The current increases with increasing bias voltage, indicating an accelerated electron transport. Hence, the enhanced driving force at −3 V during sodiation can be ascribed to the elevated electronic transport capability. The length of the amorphous Na–Se*_x_* S*_y_* region is shown in Figure 2e. The sodiation process with a larger bias voltage of −3 V results in a longer amorphous length compared to that of −1 V. In view of these results, it can be concluded that an improved electronic conductivity by increasing voltage promotes the sodiation kinetics.

**Figure 3:**
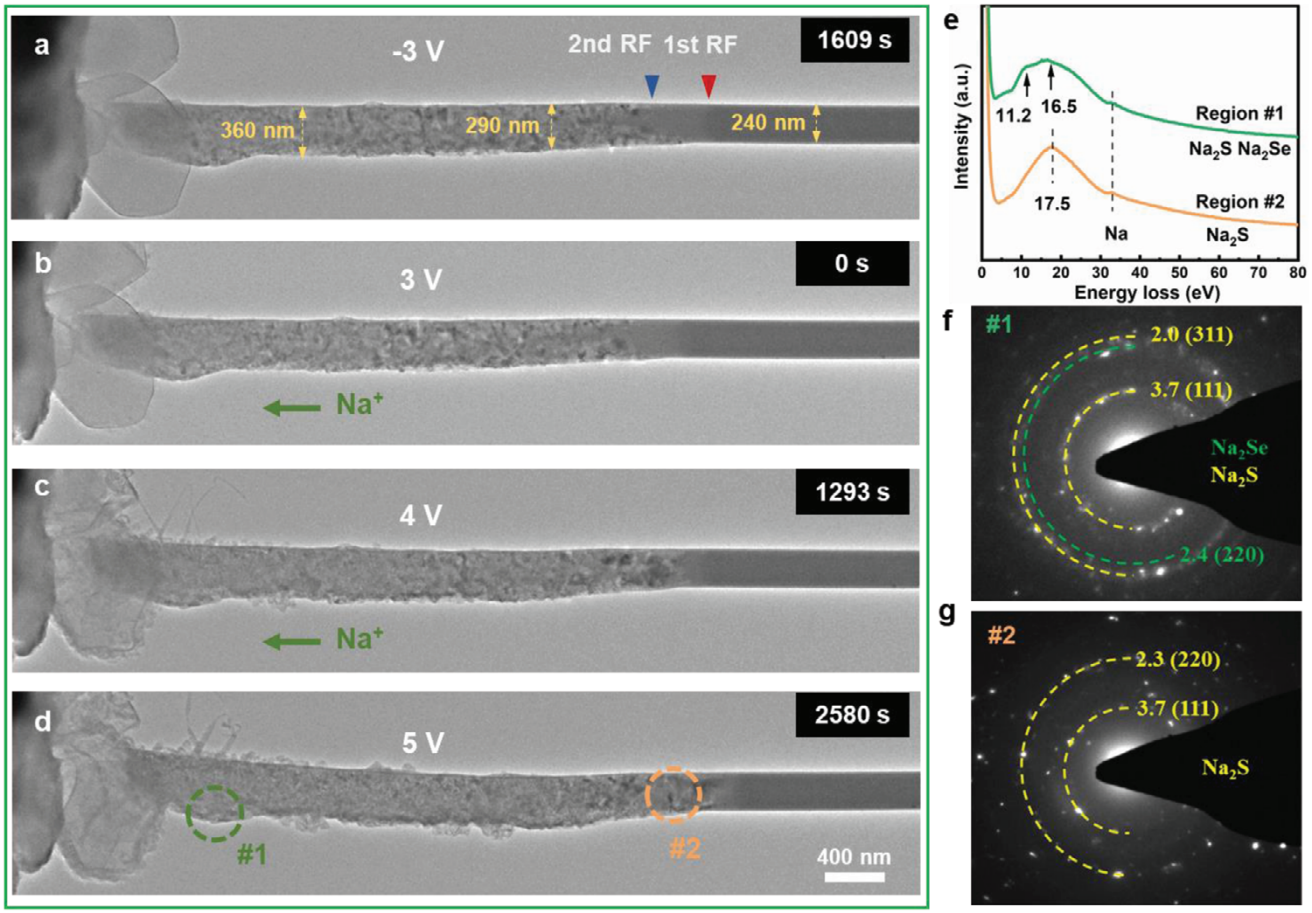
a) Time‐elapsed structural evolution of SeS_2_@CNT after full sodiation at −3 V, followed by the desodiation process with at 3 V b), 4 V c), and 5 V d) at room temperature. e) EELS profiles detected at region #1 and #2 labeled in figure d), and f,g) the corresponding EDPs.

Different from the soidation (reduction) process, the desodiation process is more complicated because of the higher energy barrier for the Na_2_S oxidation. Two approaches including an increase of the bias voltage and temperature were adopted to systematically investigate the desodiation process. First, the Na–SeS_2_ nanobattery was discharged at a voltage of −3 V and sustained for 118, 408, 910, and 1609 s (**Figure** **3**a; and Figure S5, Supporting Information). The scenario during the sodiation process is consistent with the aforementioned results. The diameter of SeS_2_@CNT (Figure 3a) changed obviously after discharging. The diameter variation and volume expansion are listed in Table S1 (Supporting Information). Subsequently, a reverse bias voltage of 3 V (Figure 3b–d) was applied at ambient temperature (24 °C) in order to extract Na ions. For convenience, the time counting was reset to 0 s. Nevertheless, the RFs are almost immobile; the morphology of SeS_2_@CNT keeps unchanged. To enable faster electron transport, higher voltages of 4 and 5 V were conducted for desodiation (Figure 3c,d). However, even after 2580 s, the morphology of SeS_2_@CNT keeps still unchanged, indicating an inhibited oxidation of Na_2_S/Na_2_Se due to the high activation energy barrier. The EELS analyses (Figure 3e) were carried out for determining the ingredient in the regions far from (#1, green circle) and near (#2, yellow circle) the 1st RF. The plasmon oscillation peaks at 11.2 and 16.5 eV for region #1 and at 17.5 eV for region #2 are assigned to Na_2_S and Na_2_Se, demonstrating a nonactivated oxidation reaction for Na_2_S and Na_2_Se. The EDPs (Figure 3f,g) captured at regions #1 and #2 confirm the unchanged Na_2_S and Na_2_Se reduction products. Consequently, even the improved electron transport capability cannot overcome the decomposition barrier of Na_2_S/Na_2_Se due to the inadequate ion transport of the nanobattery.

**Figure 4:**
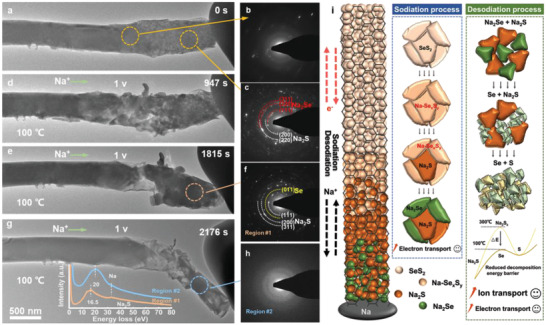
a,d,e,g) Time‐elapsed structural evolution of SeS_2_@CNT after full sodiation at −1 V a), followed by desodiation at 100 °C for different times d,e,g), and b,c,f,h) the corresponding EDPs at the selected regions. The inset of Figure f shows the EELS profiles at region #1 and #2. i) Schematic diagram illustrating the conversion reaction processes in Na–SeS_2_ nanobattery.

Generally, a higher temperature enable faster ion transport. The desodiation of Na_2_S and Na_2_Se was thus investigated at different temperatures. The presodiation on the Na–SeS_2_ nanobattery was performed at −1 V for 2364 s, as shown in Figure S6 (Supporting Information). After that, the discharged nanobattery (**Figure** **4**a) is ready for desodiation. The corresponding EDPs at different locations after sodiation are indexed to the amorphous Na–Se*_x_* S*_y_* intermediate phase (Figure 4b) and Na_2_S/Na_2_Se (Figure 4c). The temperature was raised from room temperature to 100 °C by 20 °C min^−1^. The distinct morphology variations occurred after desodiation at 100 °C (Figure 4d). An obvious volume shrinkage is observed after desodiation for 1815 s (Figure 4e) induced by the decomposition of Na_2_Se. The first appearance of Se observed from the EDP analysis (Figure 4f) indicates that Na_2_Se has a lower decomposition barrier than Na_2_S. The EELS analysis at region #1 (inset of Figure 4g) confirms the existence of Na originated from Na_2_S. When the total desodiation time reached 2176 s (Figure 4g), the nanocrystalline disappeared with a severe volume shrinkage. The EDP and EELS characterizations were carried out to figure out the oxidation products. Only amorphous halos (Figure 4h) are observed from the diffraction pattern. In addition, no Na signal is detected from the EELS analysis at region #2 (inset of Figure 4g), indicating that Na_2_Se and Na_2_S are fully decomposed and Na ions migrates back. That is, the final desodiation product are Se and S rather than SeS_2_, in agreement with the result reported previously.^[^ ^17^ ^]^ The low decomposition temperature at 100 °C demonstrates that the oxidation energy barrier of the ASS Na–SeS_2_ battery is significantly reduced than that of the Na–S system, of which the reversible oxidation needs a considerably higher temperature close to 300 °C.^[^ ^12^ ^]^ Additionally, the oxidation of Na_2_Se is prior to that of Na_2_S. Consequently, the lowered reversible oxidation temperature (only at 100 °C) for Na–SeS_2_ compared with that for Na–S ASS battery can be attributed to the existence of Se, which can provide an enhanced ionic conductivity for lowering the decomposition barrier of Na_2_S.

Based on the above‐mentioned in situ TEM analyses, several conclusions can be given. First, elevating the electronic transport capability facilitates the sodiation process, but contributes little for the oxidation of Na_2_S and Na_2_Se. In contrast, elevating the ionic transport capability attains the decomposition of Na_2_S and Na_2_Se, demonstrating that the sluggish ion transport is the dominated factor to hamper the oxidation of Na_2_S and Na_2_Se. Second, the sodiation process of the ASS Na–SeS_2_ nanobattery follows the sequence of SeS_2_→amorphous Na–Se*_x_* S*_y_* →Na_2_S and finally Na_2_S/Na_2_Se. That is, the crystalline Na_2_S is generated from amorphous Na–Se*_x_* S*_y_*, but not from SeS_2_ directly, nor from sodium polysulfides. Third, the decomposition temperature of Na_2_S for the Na–SeS_2_ nanobattery is intensively reduced from ≈300 to 100 °C, which is attributed to the lowered decomposition energy barrier by Se doping. The corresponding conversion processes are schematically displayed in Figure 4i. As a result, we propose that the improved ion transport by Se incorporation plays a dominated role on catalyzing the reversible conversion reactions in ASS Na–S batteries.

**Figure 5:**
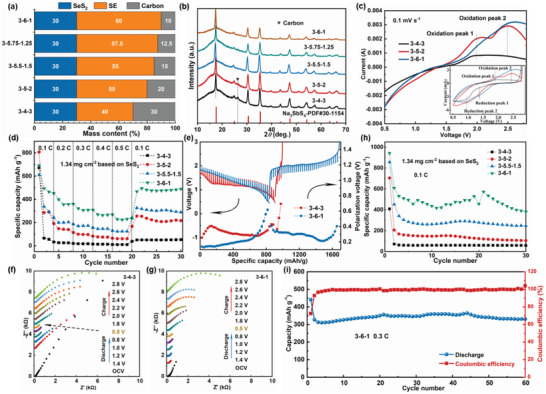
a) A bar plot of the specific mass fractions for the SeS_2_ active material, the SE, and C for different cathodes. For convenience, the samples were designated according to the ratios of SeS_2_–SE‐C. b) XRD patterns of the different cathodes. c–e) CV curves, rate performance, and GITT profiles for the selected cathodes. f,g) In situ EIS for 3‐4‐3 f) and 3‐6‐1 g). h) Cycling performance of the selected cathodes. i) Long‐term cycling performance of the ASS battery using the 3‐6‐1 cathode at a current density of 0.3 C.

The above conclusions can be used to guide the design of the composite cathodes in ASS batteries, which in turn can verify the correctness of the results based on the nanobatteries. Accordingly, the electronic and ionic conductivities were involved into the fabrication of composite cathodes. The composite cathodes with different *m*(SE)/*m*(C) ratios were investigated. *m*(SE)/*m*(C) represents the mass ratio of the solid electrolyte (SE) to carbon additive (C). The values for each component and corresponding designations (according to active material‐electrolyte‐carbon) were summarized in **Figure** **5**a. The mass fraction of the SeS_2_ active material was fixed to 30 wt%. The *m*(SE)/*m*(C) ratios are in the range of 1.33–6, enabling various ionic and electronic conductivities for the composite cathodes. The X‐ray diffraction (XRD) patterns of the cathodes are shown in Figure 5b. The patterns for all samples are dominated by the Na_3_SbS_4_ phase. The diffraction peak at 26.4° indexed to carbon is observed in 3‐4‐3 and 3‐5‐2, because of their relatively high carbon‐additive contents.

To analyze the reversibility of the discharge products, cyclic voltammetry (CV) tests were carried out for the 3‐4‐3, 3‐5‐2, 3‐6‐1 composite cathodes in a voltage window of 0.5–2.8 V with a scan rate of 0.1 mV s^−1^, as shown in Figure 5c. Two oxidation peaks located at 1.98 and 2.49 V can be ascribed to the decomposition of Na_2_Se and Na_2_S, respectively. Obviously, the oxidation currents at these peaks increase with increasing the SE fraction in the cathode. It is notable that the oxidation peaks are very weak for 3‐4‐3, indicating that a high C (30 wt%) and low SE content (40 wt%) cannot overcome the energy barrier to realize the reversibility of the active material. This confirms the in situ TEM results that the improvement of ion transport can effectively accelerate the decomposition of Na_2_S and Na_2_Se, promoting the utilization of the active materials. That is, inadequate ion transport in the composite cathode would result in impeded oxidation of Na_2_S/Na_2_Se.

The rate performance for different cathodes was conducted with current densities of 0.1, 0.2, 0.3, 0.4, 0.5 C, and then set back to 0.1 C, as shown in Figure 5d. The C rates were calculated based on the theoretical capacity of SeS_2_ (1342 mAh g^−1^). The 3‐6‐1 battery delivers the highest reversible discharge capacity (494 mAh g^−1^), while 3‐4‐3 shows almost no reversible capacity. For further exploration of the reason for the difference, the representative 3‐4‐3 and 3‐6‐1 batteries were selected for the galvanostatic intermittent titration technique (GITT) tests, as illustrated in Figure 5e. The GITT discharging profiles of 3‐6‐1 possess a higher discharging plateau and a smaller polarization voltage compared to those of 3‐4‐3, implying improved reaction kinetics for the former. In the charging process, the polarization voltages of 3‐6‐1 are below 0.4 V. In sharp contrast, the polarization voltages of 3‐4‐3 rapidly increase, indicating that the Na_2_S and Na_2_Se discharging products cannot be decomposed because of an insufficient ionic conductivity. The hindered ion transport for the 3‐4‐3 cathode is confirmed by the in situ electrochemical impedance spectra (in situ EIS), as shown in Figure 5f,g. At the initial state, 3‐4‐3 shows a larger diffusion resistance in contrast to 3‐6‐1, demonstrating an inadequate ionic conductivity within the cathode. After discharging to 0.5 V, 3‐4‐3, and 3‐6‐1 show similar diffusion resistances due to the formation of Na_2_Se/Na_2_S.

The ASS batteries assembled with different cathodes (Figure 5h) were tested at 0.1 C to further compare the electrochemical performance. The loading of the SeS_2_ active material is 1.34 mg cm^−2^. At the second cycle, the 3‐4, 3‐5‐2, 3‐5.5‐1.5, and 3‐6‐1 batteries deliver discharge capacities of 71, 205, 451, and 608 mAh g^−1^, respectively. Even subjected to a higher current density of 0.3 C, the 3‐6‐1 battery still delivers a capacity of ≈330 mAh g^−1^ with good cycling retention, as shown in Figure 5i. The 3‐6‐1 battery was further assembled with a high area loading of 5.1 mg cm^−2^ based on SeS_2_ (Figure S7, Supporting Information). It also exhibits a relatively high reversible capacity of 469 mAh g^−1^ at 0.05 C with good cycling stability. Albeit SeS_2_ transforms irreversibly to Se and S at the first desodiation according to the in situ TEM result, it would not affect the cycling performance. This is because the transition enables the activation of S for participating the electrochemical reaction with Na and, the amounts of Na involved in the conversion reaction are not affected. In parallel, the optimization of the cathode constitution instructed by the in situ TEM result improves the ion transport ability in the composite cathode. Thus, the 3‐6‐1 battery achieves a decent capacity, high Coulombic efficiency, and good cycling stability. This result also demonstrates that a higher electrolyte fraction with an enhanced ionic conductivity is beneficial to better utilize the active materials via promoting the oxidation of Na_2_S/Na_2_Se, in accordance with the TEM results. In view of this point, it is significant to develop new electrolytes with the high ionic conductivity and properly design the ion conducting network within the composite cathode.

To gain a deeper insight into the relationship between the ion/electron transport and the fraction of the conducting phases, the quantitative transport analyses for the composite cathodes in ASS Na–SeS_2_ batteries were systematically investigated. The effective transport properties for various component proportions in the cathode were evaluated through the direct current (DC) polarization method, which is relevant to the volume fractions of SE (*ϕ*(SE)) and C (*ϕ*(C)) in the composite cathode. The volume fractions were calculated from the theoretical density and mass fraction of each component, which are listed in Table S2 (Supporting Information). The effective total conductivity (*σ*_eff_) is related to the conductivity of SE or carbon (*σ*_0_), the volume fraction (*ϕ*), and the tortuosity factor (*τ* ^2^) of transport pathways in the composite cathode^[^ ^18^ ^]^

(1) $$\sigma_{\mathrm{eff}}=\frac{\phi }{\tau^{2}}\;\cdot \sigma_{0}$$

**Figure 6:**
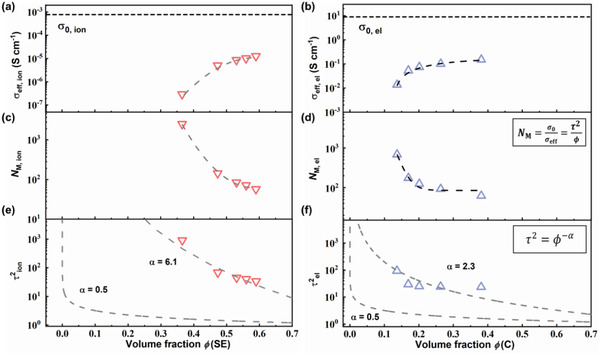
a,b) Effective ionic a) and electronic b) conductivity of the composite cathodes. c–f) MacMullin number *N*_M_ c,d) and tortuosity factors *τ* ^2^ e,f) of the ion and electron transport.

Herein, the effective total ionic conductivity (*σ*_eff, ion_) and the effective total electronic conductivity (*σ*_eff, el_) were measured from the composite cathodes, as shown in Figure S8 (Supporting Information). The ionic conductivity of Na_3_SbS_4_ (*σ*_0, ion_) was measured using EIS (Figure S9, Supporting Information). The electronic conductivity of C (*σ*_0, el_) was measured using the DC polarization method (Figure S10, Supporting Information). Please be noted that Na_3_SbS_4_ and C can be assumed as pure ionic and electronic conducting phase, respectively, as Na_3_SbS_4_ has a negligible electronic conductivity of 1.78 × 10^−8^ S cm^−1^ (Figure S11, Supporting Information). The *σ*_eff, ion_−*ϕ*(SE) and *σ*_eff, el_−*ϕ*(C) curves are, respectively, shown in **Figure** **6**a,b. The dotted lines in the figure guide the *σ*_0, ion_ (0.72 mS cm^−1^) and *σ*_0, el_ (9334 mS cm^−1^) values. *σ*_eff, ion_ increases almost linearly with increasing *ϕ*(SE), indicating faster ion transport regarding a higher *ϕ*(SE). By contrast, the increase of *σ*_eff, elec_ becomes very slow when *ϕ*(C) higher than 0.2, indicating that a low volume fraction of C is enough to meet the demand for constructing effective electronic pathways.

The MacMullin number *N*_M_^[^ ^19^ ^]^ was introduced to further compare the effective electron and ion transport performance with the formula

(2) $$N_{M}=\frac{\sigma_{0}}{\sigma_{\mathrm{eff}}}\;=\frac{\tau^{2}\;}{\phi }\;$$

A larger *N*_M_ thus indicates severer impeded transport in the cathode. As shown in Figure 6c,d, the *N*_M_ values of the ion transport (*N*_M, ion_) are in a wide range of 57–2490, significantly broader than those of electron transport (*N*_M, el_, 62–681). This demonstrates that the ion tranport is the bottleneck limiting the cathode performance, because good electron conducting networks can be constructed by a small amount of carbon additives. Generally, the *N*_M, ion_ values for a liquid electrolyte are below 20, as liquid can infiltrate the voids among the electrodes.^[^ ^20^ ^]^ However, the solid–solid point contacts in ASS batteries cause a large amount of voids even pressed under a high pressure. This is another reason to make it a challenge to construct a good ion conducting network in ASS batteries.

The tortuous degree of the the charge transport in a composite cathode should be also taken into account for the compositional design. To quantify the tortuous degree, the Bruggeman model is introduced as follows^[^ ^18^, ^21^ ^]^

(3) $$\tau^{2}=\phi^{-\alpha }\;$$

The tortuosity factor *τ* ^2^ is inversely correlated to the volume fraction. If *α* = 0.5, the inclusions that hinder carrier transport are defined as a spherical model, as marked with the dot dash lines in Figure 6e,f. The relations between *α* values and geometry of the particles are listed in Table S3 (Supporting Information). However, the morphologies of the electron/ion conducting phases in the cathode are irregular. The *α* values for the ion and electron transport was respectively calculated as 6.1 and 2.3, demonstrating high tortuosities and a more constricted ion transport pathway compared with the electron transport pathway. All the values of *σ*_eff_, *N*_M_, and *τ* ^2^ for various components are summarized in Table S4 (Supporting Information). The above results indicate that a high fraction of SE additive in the composite cathode is requisite to provide sufficient ion transport pathways due to the relatively low ionic conductivity and high tortuosity factor of the SE. Meanwhile a trace of carbon is necessary to allow effective electron transport. Albeit the *α* values only roughly evaluate the tortuosity of the charge transport, they effectively give the information that the ions migrate through more tortuous pathways. Thus, the measurement of *α* values can identify the bottleneck of the charge transport in the cathode.

## 3 Conclusions

In summary, to reveal the effect of Se incorporation in S‐based active material, the sodiation and desodiation processes of Na–SeS_2_ nanobattery were systematically investigated using in situ TEM with EDP and EELS. An amorphous Na–Se*_x_* S*_y_* is found for the first time to exist as an intermediate phase in the sodiation process, which significantly lowers the redox reaction barrier. Thus, we proposed the Se incorporation in SeS_2_ enables the direct formation of Na_2_S/Na_2_Se from Na–Se*_x_* S*_y_* via the sodiation sequence: SeS_2_→amorphous Na–Se*_x_* S*_y_* →Na_2_S and finally Na_2_S/Na_2_Se. The intermediate phase is markedly distinct from the Na–S nanobattery where the intermediates are polysulfides. During the desodiation process, the oxidation of Na_2_S/Na_2_Se was obstructed due to the inadequate ion transport in the nanobattery, even with the improved electronic conductivity. The successful desodiation of Na_2_S/Na_2_Se is attained at 100 °C, where the ion transport is facilitated. The decomposition of Na_2_Se forms Se and is prior to the decomposition of Na_2_S. Compared with the Na–S nanobattery with a desodiation temperature of 300 °C, the lowered temperature of 100 °C in the Na–SeS_2_ nanobattery evidenced an intensively reduced decomposition energy barrier by the Se incorporation. We thus conclude that the formation of the Na–Se*_x_* S*_y_* intermediate phase in the sodiation process and the incorporation of Se can effectively catalyze the reversible redox reaction in the Na–S batteries. These findings are applicable to other S‐based active materials.

Guided by the in situ TEM results, ASS SeS_2_ batteries were assembled using various SE:C ratios in the composite cathodes to investigate the effect of ion and electron transport on the electrochemical properties. The CV, GITT, and the in situ EIS results in turn confirm the conclusion obtained from the in situ TEM. Additionally, the evaluation on the effective transport performance, the MacMullin number *N*_M_, and the tortuosity factor *τ* ^2^ for different SE:C ratios demonstrate that the electron transport is easy to reach a saturated state with increasing the C content, while the ion transport is continuously enhanced with increasing the SE content, indicating that the use of SE additive with a high ionic conductivity in the composite cathode is advantageous. Fast ion transport are thus the key factor to achieve a good rate capability and decent capacity and cycling performance. Our discovery provides a reasonable optimization strategy toward Na–S battery.

## 4 Experimental Section

### Sample Preparation

The preparations of composite cathodes are as follows. The Na_3_SbS_4_ SE was prepared using a solid‐state reaction, as described in the early work.^[^ ^22^ ^]^ The Na_3_SbS_4_ SE, SeS_2_ power (Aladdin 97%), and the C additive (acetylene black) were used for preparing the composite cathodes. The three materials with a weight of 0.5 g were mixed in a ball milling device (Fritsch Pulverisette 7) with a rotation speed of 200 rpm for 2 h, followed by a rotation speed of 500 rpm for 1 h. The component design of the composite cathodes is listed in Table S2 (Supporting Information).

The preparation of SeS_2_@CNT refers to Huang’ s method.^[^ ^23^ ^]^ Initially, an anodic aluminum oxide (AAO) template with the pore size of about 200 nm was heated to 700 °C for depositing a carbon layer with a thickness about 15 nm via C_3_H_6_ decomposition. Then the carbon‐coated AAO was submerged in a molten selenium sulfide under 150 °C for the percolation of selenium sulfide into the carbon‐coated AAO. The exceed selenium sulfide was removed by elevating the temperature to 250 °C. Finally, the SeS_2_@CNT was obtained by etching the AAO template with hydrofluoric acid.

### Material Characterization

XRD was conducted using Rigaku D/MAX‐2500/PC (Cu K *α*, 40 kV 200 mA) in the range of 10°–70°. In situ TEM characterizations were carried out in a Cs‐corrected TEM (FEI, Titan G2, 300 kV). The electrochemical properties of the ASS batteries were studied on a Neware system. The cyclic voltammetry (CV) tests were conducted on a Princeton 4000 electrochemical workstation by applying a scan rate of 0.1 mV s^−1^ in the voltage range of 0.5–2.8 V. The GITT tests were charged/discharged at a current density of 0.017 C for 1 h then resting for 2 h. The in situ EIS were conducted in a frequency range of 1 Hz to 5 MHz at voltages of 1.4, 1.2, 1.0, 0.8, and 0.5 V during discharging process and 1.8, 2.0, 2.2, 2.4, 2.6, and 2.8 V during charging process at room temperature.

### DC Polarization Test

The electron‐ and ion‐blocking cells for DC polarization tests were respectively assembled by using the multilayer Na_15_Sn_4_ǀǀNa_3_SbS_4_ǀǀSeS_2_‐SE‐CǀǀNa_3_SbS_4_ǀǀNa_15_Sn_4_ and steelǀǀSeS_2_‐SE‐Cǀǀsteel symmetric cells, where Na_3_SbS_4_ is an electronic insulator. For assembling process of the former, 40 mg composite cathode was first pressed in a home‐made device (7.5 mm diameter) at 100 MPa, then 40 mg Na_3_SbS_4_ was pressed on both sides of the composite cathode at 200 MPa. Finally, 30 mg Na_15_Sn_4_ was pressed on both sides of the as‐prepared Na_3_SbS_4_ǀǀSeS_2_‐SE‐CǀǀNa_3_SbS_4_ cell at 300 MPa. For the latter, SeS_2_‐SE‐C was pressed at 300 MPa and the stainless steel foils were attached on both sides to block the ions.

### The Construction of Nanobatteries and ASS Batteries

The nanobattery setup consists of a SeS_2_@CNT working electrode, metallic Na counter/reference electrodes, and Na_2_O SE naturally formed on the Na surface. For assembling, SeS_2_@CNT was dispersed in ethanol, then dropped to an aluminum rod and dried. The metallic Na was scratched onto another side of the aluminum rod. The assembled nanobatteries were constructed on a microelectrochemical systems (MEMS) heating device, which comprises of an electric circuit and a heating circuit. Subsequently, the device was inserted into a Cs‐corrected environmental TEM (FEI, Titan G2, 300 kV) for observation.

The ASS Na–SeS_2_ batteries were assembled in a home‐made mold with a diameter of 10 mm. The as‐prepared composite cathodes with an area loading of 1.34 mg cm^−2^, Na_3_SbS_4_ electrolyte (100 mg), and Na_15_Sn_4_ (50 mg) powders were cold‐pressed under a pressure of 350 MPa for rate and cycling characterizations.

### Fitting Analysis

The Origin software (OriginLab trail version) was utilized for data fitting/analyzing. Due to the heavy workload for sample preparations, only one sample (composite cathode) was prepared for each data point to get a rough comparison. Five data points were collected for each fitting. For all fitting, the *R* ^2^ values exceed 0.98, and the *p* values are less than 0.05, indicating a valid fitting.

## Conflict of Interest

The authors declare no conflict of interest.

## Supporting information

Supporting information

## Data Availability

Research data are not shared.

## References

[1] N. Kittner , F. Lill , D. M. Kammen , Nat. Energy 2017, 2, 17125.

[2] a) Z. Yan , Y. Liang , J. Xiao , W. Lai , W. Wang , Q. Xia , Y. Wang , Q. Gu , H. Lu , S. L. Chou , Y. Liu , H. Liu , S. X. Dou , Adv. Mater. 2020, 32, 1906700; 10.1002/adma.201906700 31943381

[3] a) Y. Qi , Q. J. Li , Y. Wu , S. J. Bao , C. Li , Y. Chen , G. Wang , M. Xu , Nat. Commun. 2021, 12, 6347; 34732738 10.1038/s41467-021-26631-y PMC8566531

[4] a) A. Banerjee , X. Wang , C. Fang , E. A. Wu , Y. S. Meng , Chem. Rev. 2020, 120, 6878; 32603100 10.1021/acs.chemrev.0c00101

[5] a) S. F. Ng , M. Y. L. Lau , W. J. Ong , Adv. Mater. 2021, 33, 2008654; 10.1002/adma.202008654 33811420

[6] a) F. Sun , H. Cheng , J. Chen , N. Zheng , Y. Li , J. Shi , ACS Nano 2016, 10, 8289; 27522865 10.1021/acsnano.6b02315

[7] X. Li , J. Liang , J. Luo , C. Wang , X. Li , Q. Sun , R. Li , L. Zhang , R. Yang , S. Lu , H. Huang , X. Sun , Adv. Mater. 2019, 31, 1808100. 10.1002/adma.201808100 30873698

[8] Z. Li , J. Zhang , H. B. Wu , X. W. D. Lou , Adv. Energy Mater. 2017, 7, 1700281.

[9] a) J. Timoshenko , B. R. Cuenya , Chem. Rev. 2021, 121, 882; 32986414 10.1021/acs.chemrev.0c00396 PMC7844833

[10] a) J. Cui , H. Zheng , K. He , Adv. Mater. 2021, 33, 2000699; 10.1002/adma.202000699 32578290

[11] a) F. A. Perras , S. Hwang , Y. Wang , E. C. Self , P. Liu , R. Biswas , S. Nagarajan , V. H. Pham , Y. Xu , J. A. Boscoboinik , D. Su , J. Nanda , M. Pruski , D. Mitlin , Nano Lett. 2020, 20, 918; 31815484 10.1021/acs.nanolett.9b03797

[12] Y. Li , Y. Tang , X. Li , W. Tu , L. Zhang , J. Huang , Small 2021, 17, 2100846. 10.1002/smll.202100846 33983675

[13] a) Y. Luo , Y. Bai , A. Mistry , Y. Zhang , D. Zhao , S. Sarkar , J. V. Handy , S. Rezaei , A. C. Chuang , L. Carrillo , K. Wiaderek , M. Pharr , K. Xie , P. P. Mukherjee , B.‐X. Xu , S. Banerjee , Nat. Mater. 2022, 21, 217; 34824396 10.1038/s41563-021-01151-8

[14] X. Yao , N. Huang , F. Han , Q. Zhang , H. Wan , J. P. Mwizerwa , C. Wang , X. Xu , Adv. Energy Mater. 2017, 7, 1602923.

[15] a) P. Minnmann , L. Quillman , S. Burkhardt , F. H. Richter , J. Janek , J. Electrochem. Soc. 2021, 168, 040537;

[16] T. Yang , Y. Qi , W. Zhong , M. Tao , B. Guo , Y. Wu , S. J. Bao , M. Xu , Adv. Funct. Mater. 2020, 31, 2001952.

[17] Y. Cui , A. Abouimrane , J. Lu , T. Bolin , Y. Ren , W. Weng , C. Sun , V. A. Maroni , S. M. Heald , K. Amine , J. Am. Chem. Soc. 2013, 135, 8047. 23631402 10.1021/ja402597g

[18] A. Bielefeld , D. A. Weber , J. Janek , ACS Appl. Mater. Interfaces 2020, 12, 12821. 32093477 10.1021/acsami.9b22788

[19] a) K. K. Patel , J. M. Paulsen , J. Desilvestro , J. Power Sources 2003, 122, 144;

[20] G. F. Dewald , S. Ohno , J. G. C. Hering , J. Janek , W. G. Zeier , Batteries Supercaps 2020, 4, 183.

[21] J. Landesfeind , J. Hattendorff , A. Ehrl , W. A. Wall , H. A. Gasteiger , J. Electrochem. Soc. 2016, 163, A1373.

[22] a) Z. Wang , L. Zhang , X. Shang , W. Wang , X. Yan , C. Yu , L.‐m. Wang , Chem. Eng. J. 2022, 428, 132094;

[23] Z. Wang , Y. Tang , L. Zhang , M. Li , Z. Shan , J. Huang , Small 2020, 16, 2001899. 10.1002/smll.202001899 32519445

