# Supplementary material for: Unraveling the Conversion Evolution on Solid‐State Na–SeS2 Battery via In Situ TEM
Source: Adv Sci (Weinh). 2022 Mar 23;9(14):2200744. doi: 10.1002/advs.202200744 (PMC9109063; doi:10.1002/advs.202200744)
Supplement: Supplementary file 1 — Supporting information [file ADVS-9-2200744-s001.pdf]

Supporting Information

Unraveling the Conversion Evolution on Solid-State Na-SeS_2 Battery via In Situ TEM

Ziqi Zhang[1#], Zaifa Wang[1#], Long Zhang[1*], Di Liu[1], Chuang Yu[2], Xinlin Yan[3], Jia Xie[2], Jianyu Huang[1]

[1]Clean Nano Energy Center, State Key Laboratory of Metastable Materials Science and Technology, Yanshan University, Qinhuangdao 066004, Hebei, China

[2]State Key Laboratory of Advanced Electromagnetic Engineering and Technology, School of Electrical and Electronic Engineering, Huazhong University of Science and Technology, Wuhan 430074, P. R. China

[3]Institute of Solid State Physics, Vienna University of Technology, Vienna 1040, Austria

#The authors make equal contribution.

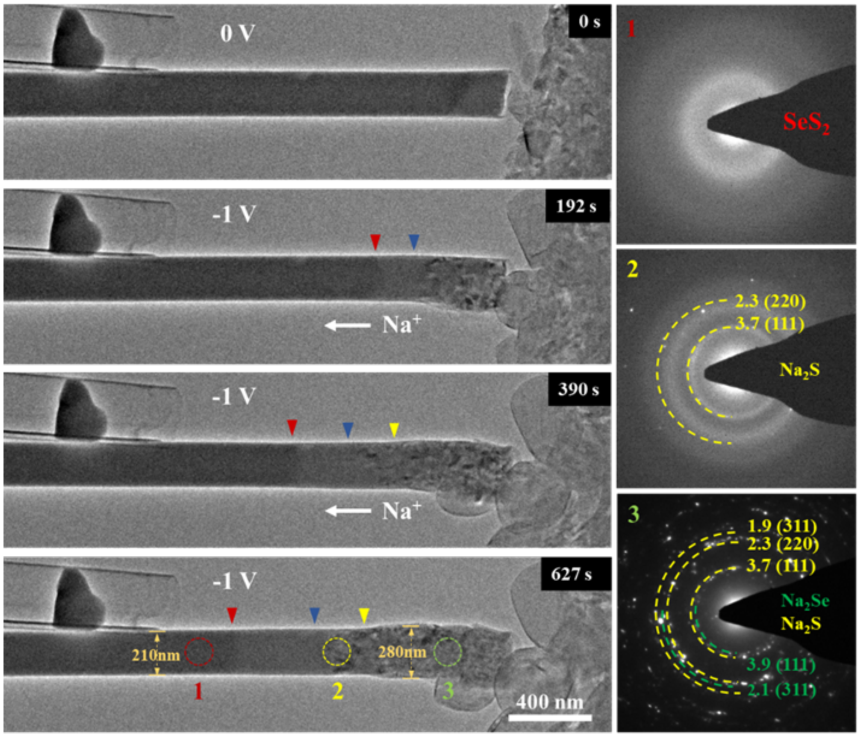

Figure S1. Time-elapsed structural evolution tested on another set of Na-SeS_2@CNT nanobattery upon sodiation under a bias voltage of -1 V, and the corresponding EDPs at the locations labeled with 1, 2, 3. This is a reproducible experiment with respect to the nanobattery shown in Figure 1.

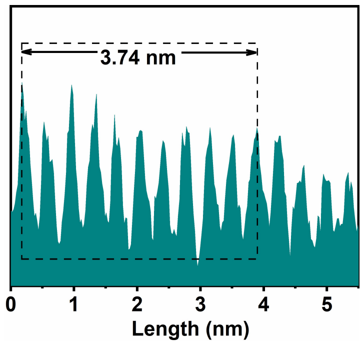

Figure S2. Crystal plane spacing obtained from the HRTEM image shown in Figure 1g.

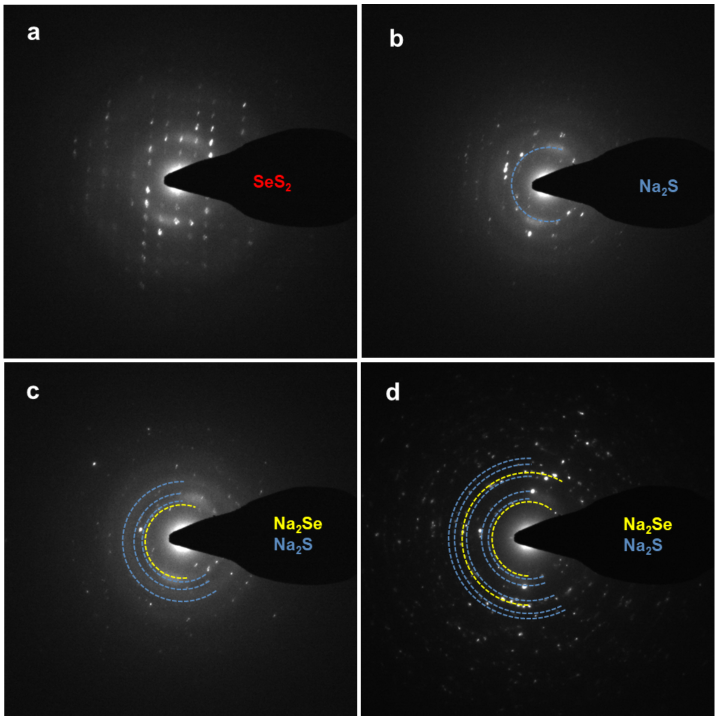

Figure S3. Operando EDPs of SeS_2@CNT upon the sodiation process. This is a reproducible test on another set of Na-SeS_2@CNT nanobattery regarding the observation shown in Figure 1i.

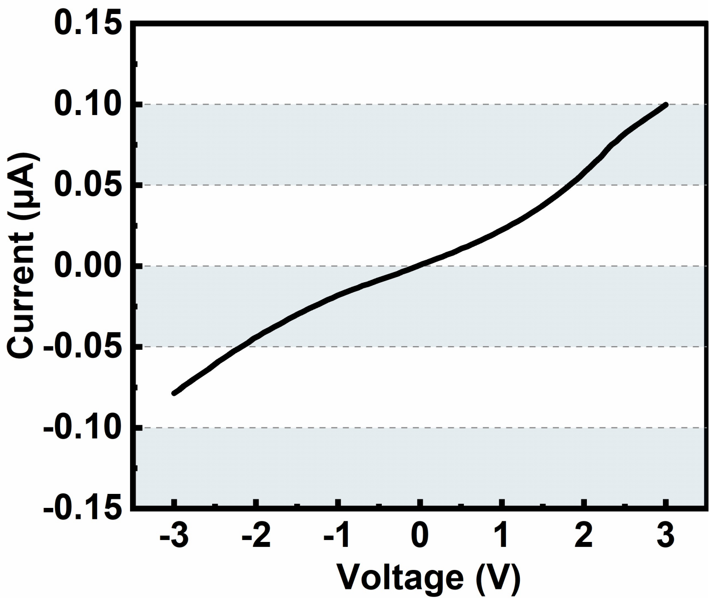

Figure S4. I–V curve applied for the in situ TEM tests on the Na-SeS_2@CNT nanobatteries.

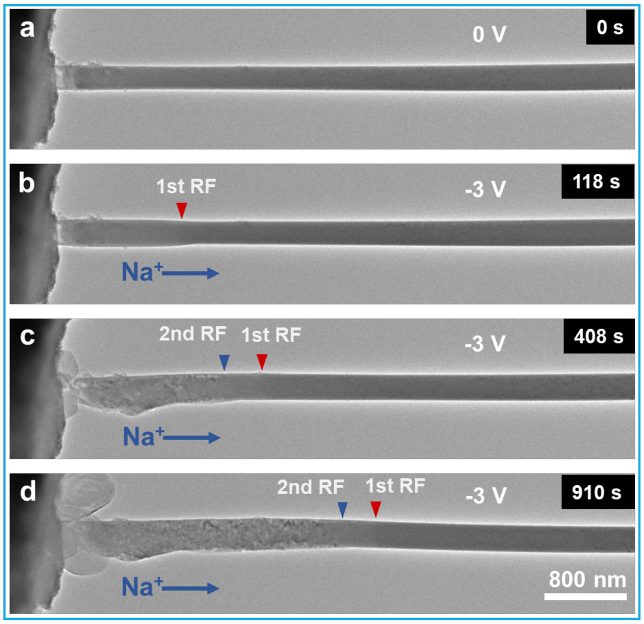

Figure S5. Time-elapsed structural evolution of SeS_2@CNT upon sodiation (a–d) with a bias voltage of -3 V for various durations.

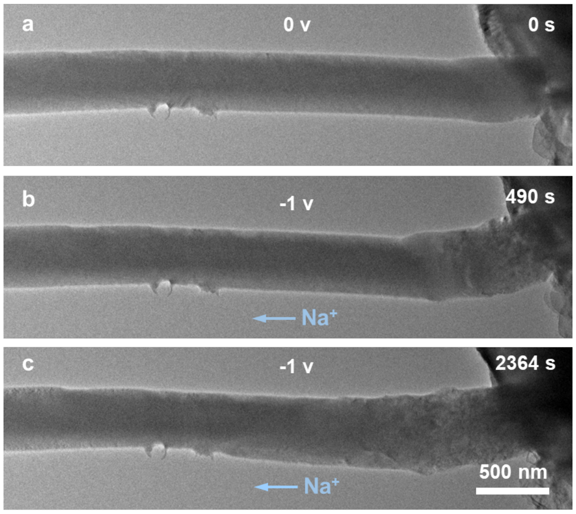

Figure S6. A presodiation on the Na-SeS_2@CNT nanobattery prepared for subsequent desodiation at 100 °C.

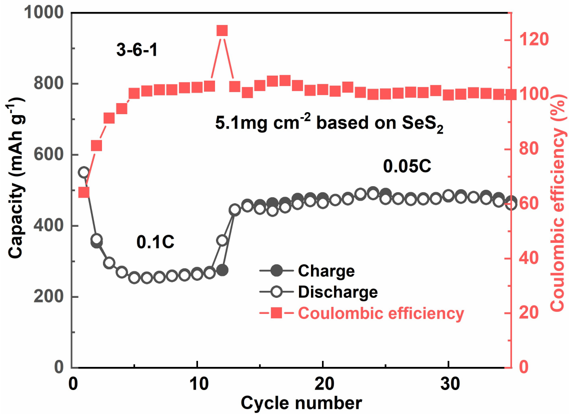

Figure S7. Cycling performance of the 3-6-1 cathode with a loading of 5.1 mg/cm^2 based on SeS_2.

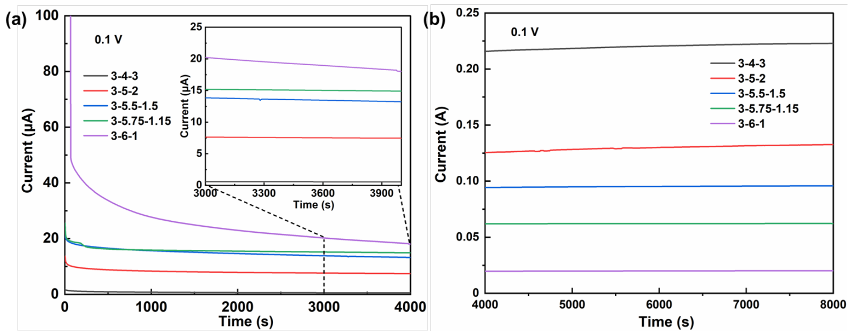

Figure S8. DC polarization curves for calculating the effective transport properties, MacMullin number N_M, and the tortuosity factor τ^2.

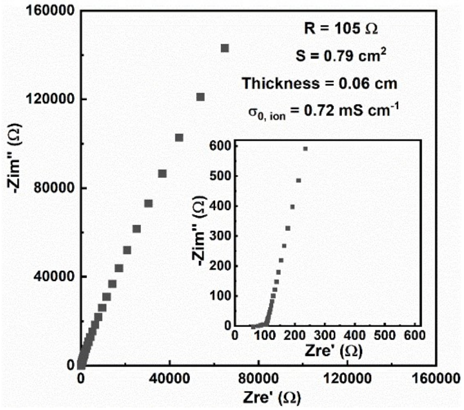

Figure S9. EIS curve of the Na_3SbS_4 SE for calculating the ionic conductivity.

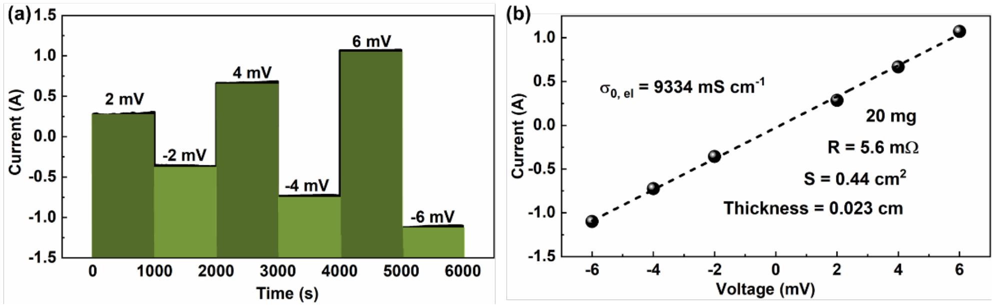

Figure S10. Electronic conductivity for the C additive used for constructing the composite cathodes.

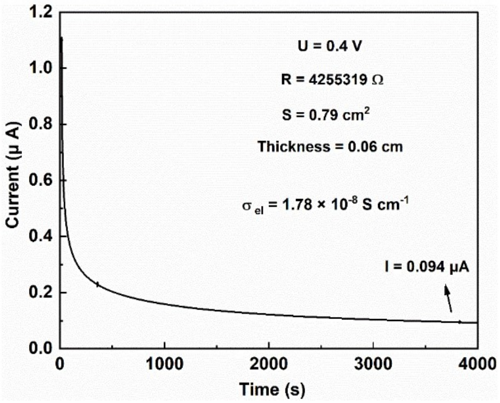

Figure S11. DC polarization curve for calculating the electronic conductivity of the Na_3SbS_4 SE.

Tabel S1. The diameter variation and the volume expansion of SeS_2@CNT for various SeS_2 at different voltages.

Sample	Initial diameter (nm)	Final diameter (nm)	Volume expansion
Single-crystal SeS_2 (-1 V)	210	310	118%
Amorphous SeS_2 (-1 V)	210	280	77%
Another single-crystal SeS_2 (-3 V)	240	360	125%

Tabel S2. The mass and volume fractions for different composite cathodes.

$m(SeS_2)$	m(SE)	m(C)	$\phi(SeS_2)$	ϕ(SE)	ϕ(C)	ϕ(SE)/ϕ(C)	ϕ(SE)/$\phi(SeS_2)$
0.300	0.400	0.300	0.254	0.365	0.381	0.959	1.439
0.300	0.500	0.200	0.263	0.473	0.263	1.799	1.799
0.300	0.550	0.150	0.268	0.531	0.201	2.638	1.978
0.300	0.575	0.125	0.271	0.560	0.169	3.309	2.068
0.300	0.600	0.100	0.273	0.590	0.137	4.317	2.158

Density used: SeS_2: 3 g/cm^3; C: 2 g/cm^3; Na_3SbS_4: 2.78 g/cm^3.

Tabel S3. The α values associated with the particle geometry.

α value	Geometry
0.5	spheres
1	cylinders

Tabel S4. The values of σ_{eff}, N_M, τ^2 for different composite cathodes.

	$\sigma_{eff, ion}$ (S/cm)	$N_{M, ion}$	$\tau^2_{, ion}$	$\sigma_{eff, el}$ (S/cm)	$N_{M, el}$	$\tau^2_{, el}$
3-4-3	2.89×10^{-7}	2489.7	909.5	1.51×10^{-1}	61.7	23.5
3-5-2	5.04×10^{-6}	142.8	67.6	1.01×10^{-1}	92.2	24.3
3-5.5-1.5	8.55×10^{-6}	84.2	44.7	7.48×10^{-2}	124.9	25.1
3-5.75-1.25	1.01×10^{-5}	71.3	39.9	5.35×10^{-2}	174.5	29.5
3-6-1	1.26×10^{-5}	57.0	33.6	1.37×10^{-2}	681.8	93.2
